# Heat and PAHs Emissions in Indoor Kitchen Air and Its Impact on Kidney Dysfunctions among Kitchen Workers in Lucknow, North India

**DOI:** 10.1371/journal.pone.0148641

**Published:** 2016-02-12

**Authors:** Amarnath Singh, Ritul Kamal, Mohana Krishna Reddy Mudiam, Manoj Kumar Gupta, Gubbala Naga Venkata Satyanarayana, Vipin Bihari, Nishi Shukla, Altaf Hussain Khan, Chandrasekharan Nair Kesavachandran

**Affiliations:** 1 Epidemiology Division, CSIR-Indian Institute of Toxicology Research, PB No 80, MG Marg, Lucknow, 226001, U.P, India; 2 Environment Monitoring Division, CSIR-Indian Institute of Toxicology Research, PB No 80, MG Marg, Lucknow, 226001, U.P, India; 3 Analytical Chemistry Division, CSIR-Indian Institute of Toxicology Research, PB No 80, MG Marg, Lucknow, 226001, U.P, India; 4 Department of Biochemistry, Babu Banarasi Das University, BBD City, Faizabad Road, Lucknow, Uttar Pradesh - 226 028, U.P, India; University of Utah School of Medicine, UNITED STATES

## Abstract

Indoor air quality and heat exposure have become an important occupational health and safety concern in several workplaces including kitchens of hotels. This study investigated the heat, particulate matter (PM), total volatile organic compounds (TVOCs) and polycyclic aromatic hydrocarbons (PAHs) emissions in indoor air of commercial kitchen and its association with kidney dysfunctions among kitchen workers. A cross sectional study was conducted on 94 kitchen workers employed at commercial kitchen in Lucknow city, North India. A questionnaire-based survey was conducted to collect the personal and occupational history of the kitchen workers. The urine analysis for specific gravity and microalbuminuria was conducted among the study subjects. Indoor air temperature, humidity, wet/ dry bulb temperature and humidex heat stress was monitored during cooking activities at the kitchen. Particulate matter (PM) for 1 and 2.5 microns were monitored in kitchen during working hours using Hazdust. PAH_S_ in indoor air was analysed using UHPLC. Urinary hydroxy-PAHs in kitchen workers were measured using GC/MS-MS. Higher indoor air temperature, relative humidity, PM_1_ and PM_2.5_ (p<0.001) was observed in the kitchen due to cooking process. Indoor air PAHs identified are Napthalene, fluorine, acenaphthene, phenanthrene, pyrene, chrysene and indeno [1,2,3-cd) pyrene. Concentrations of all PAHs identified in kitchen were above the permissible OSHA norms for indoor air. Specific gravity of urine was significantly higher among the kitchen workers (p<0.001) as compared to the control group. Also, the prevalence of microalbuminuria was higher (p<0.001) among kitchen workers. Urinary PAH metabolites detected among kitchen workers were 1-NAP, 9-HF, 3-HF, 9-PHN and 1-OHP. Continuous heat exposure in kitchens due to cooking can alter kidney functions *viz*., high specific gravity of urine in kitchen workers. Exposure to PM, VOCs and PAHs in indoor air and presence of urinary PAHs metabolites may lead to inflammation, which can cause microalbuminuria in kitchen workers, as observed in the present study.

## Introduction

Environments with high heat and humidity, such as kitchens, contribute to heat-related illnesses among workers who spend long hours under these stress conditions. Heat stress in commercial kitchens can be due to high air temperature, humidity, radiant heat and airflow [1]. Thermal strain induced by such occupations can lead to health risks like acute heat stroke, blood circulation problems, and discomfort at work place [2]. Heat stress is still the most neglected occupational hazard in tropical and subtropical countries [3]. Heat stress and related dehydration can result in acute or chronic kidney failure [4]. Depletion of water and sodium correlates with adverse microalbuminuria [5], which in turn has been considered as an early surrogate marker for kidney damage [6].

An earlier study demonstrated that kitchen workers in gas kitchens were subjected to a higher thermal strain than those in electric kitchens in Japan [7]. Cooking fumes, especially from frying contain fine particles (Particulate matter <2.5 μm size) and ultrafine particles (UFP) [8–12]. The different chemical substances identified in cooking fumes include aldehydes, polycyclic aromatic hydrocarbons (PAHs), heterocyclic amines, aromatic amines, and alkanoic acids [8, 11–14]. Personal measurements performed during the frying of beefsteak on both gas and electric stoves have shown that cooks are exposed to PAHs, though in low concentrations [12, 15].

Except for cooking practice, cooking oil-fumes should make a significant contribution to PAHs concentrations of indoor air including two parts: (1) once heated PAHs evaporate from the polluted oil into air; (2) at high temperature organic compounds are partially cracked to smaller unstable fragments (pyrolysis), mostly radicals recombine to give relatively stable PAHs (pyrosynthesis) [16]. Epidemiological studies show an elevated incidence of cancers among non-smoking women with long-term exposure to cooking oil-fume [17, 18] and an excessive bladder cancer rate among cooks exposed to kitchen air [19].

As per the earlier report [20] “Measuring urinary metabolites as biomarkers for human exposure assessment has the following advantages: (i) urine samples are readily available and noninvasive; (ii) the method provides direct evidence of human exposure to carcinogens; and (iii) the data represent integrated exposure via multiple media over a period of time [20]. Urinary hydroxylated PAH metabolites have been measured in occupationally exposed populations, including coke oven workers, aluminum smelter workers, foundry workers and road pavers [21–25]. Urinary 1-hydroxypyrene was used as a biomarker to investigate the half-life of PAH compounds in study subjects consuming charcoal-grilled meat [26].

Kitchen workers are prone to heat stress at work place due to heat generated from cooking practices especially in tropical countries like India. Multiple exposure of PM, PAHs and heat exposure in indoor air and their association on kidney dysfunctions like microalbumiuria among kitchen workers has not been investigated earlier. Hence, a preliminary investigation was undertaken to find out the association between indoor air pollutants and kidney dysfunctions among kitchen workers.

## Methods

### Chemical and Reagents

All chemicals and reagents were used in this study were analytical grade unless otherwise stated. The 16 PAHs [Naphthalene (NA), Acenaphthene (ACEN), Acenaphthylene (ACY), Fluorene (FLUOR), Phenanthrene (PHEN), Anthracene (AN), Fluoranthene (FLUR), Pyrene (PY), Benzo[a]Anthracene (BaA), Chrysene (CHRY), Benzo[b]-fluoranthene (BbF), Benzo[k]-fluoranthene (BkF), Benzo[a]-pyrene (BaP), Indeno[1,2,3-cd]pyrene (Ind), Dibenzo[a,h]-anthracene (DBA) and Benzo[ghi]perylene (BPe)] and selected PAH metabolites viz., 1-napthol (1-NAP), 9-phenanthrol (9-PHN), 1-hydroxypyrene (1-OHP), 2-hydroxyfluorene (2-HF),3-hydroxyfluorene (3-HF), 9-hydroxyfluorene (9-HF) and bis(trimethylsilyl)trifluoroacetamide with 1% trimethylchlorosilane (BSTFA & TMCS; 99:1; *v/v*), sodium hydroxide (NaOH), potassium hydroxide (KOH) were procured from Sigma (St Louis, MO, USA). Acetonitrile (ACN), methanol (MeOH), ethanol (EtOH), trichloroethylene (TCE) and *n*-hexane (HEX) were purchased from Merck (Darmstadt, Germany). Ultrapure water was produced by the Milli-Q water purification system (Millipore, Badford, MA, USA). Individual stock solution of each PAH and their selected metabolites were prepared at a concentration of 1 mg/mL in ACN. All stock and standard solutions were stored at 4°C before its use for analysis.

### Study design and subjects

A cross sectional study was conducted among male kitchen workers in a city located in North India (Lucknow). Study subjects include 94 male kitchen workers involved in food preparation activities at commercial kitchen in Lucknow, North India. 94 male workers who were not involved in cooking or any kitchen-related work in commercial kitchen were selected as their corresponding controls. The control group consisted of office workers and service staff from the same institution with same socio economic status.

Both kitchen workers and control group subjects work between 09.00–17.00 hrs per day. A temporary health and environmental laboratory was set up within the premises of commercial kitchen to assess the kidney functions among kitchen workers and indoor air quality in the kitchen. The inclusion criteria for the kitchen workers were: 18–70 years of age, at least three-year work experience in kitchen, and absence of any communicable diseases during the study period. Further, kitchen workers who were smokers, consuming alcohol, caffeine, vitamin supplements or medicines like psychotropic drugs, antihypertensive drugs and antihistamines were excluded from the study, as these chemicals can inhibit thermoregulation and interfere with the study. The inclusion criteria for the control subjects were: 18–70 years of age, at least three-year work experience in non-kitchen area (office room), and absence of any communicable diseases during the study period. Further, control workers who were smokers, consuming alcohol, caffeine, vitamin supplements or medicines like psychotropic drugs, antihypertensive drugs and antihistamines were excluded from the study, as these chemicals can inhibit thermoregulation and interfere with the study. The exposed and control subjects not fulfilling these criteria were excluded from the study. Participants who gave written informed consent to participate in the study were included and those who were absent from work on the dates of the survey were excluded.

In this study, we invited 200 kitchen workers and other staff (control group) to participate. Among them, 4 kitchen workers and 3 controls refused to participate, and 2 kitchen workers and 3 controls did not provide urine samples. Therefore, 188 subjects were enrolled including 94 kitchen workers and 94 controls. No information was obtained from the 12 subjects who refused to participate. There were no significant differences in demographic characteristics between the participating subjects and the 6 kitchen workers and 6 controls who did not provide urine samples.

### Data collection

A questionnaire was prepared by the authors, and amended on the basis of the subjects’ reactions to questions following a pre-test in the field. Demographic factors such as age, sex, socio-economic status, level of education, income level, employment details, and number of working hours in the kitchen were collected and documented in a pre-tested questionnaire. Kitchen workers were monitored regularly during the working hours to ascertain how much water they were drinking.

### Ethics

Written consent was obtained from each subject after explaining the purpose of the study to subjects and satisfactorily clarifying their queries through a study brochure in Hindi (local language) and English. Ethical clearance for the study was obtained from the Institutional Human Ethics Committee- Indian Institute of Toxicology Research, Govt. of India, Lucknow, India (the committee follows the Indian Council of Medical Research ethical guidelines for biomedical research on human participants, 2006).

### Sampling sites and hygiene practices

The indoor air quality monitoring was conducted in the selected commercial kitchen during working hours of kitchen workers and other staff (9.00–17.00 h) for single day and study period was during winter in December 2014. The profile details of the sampling location (commercial kitchen) include gas stoves with efficient range hoods, windows and a no-smoking zone. During air sampling in the kitchen, the doors and windows of the kitchens were closed. This will ensure indoor air environment in kitchen and avoid pollution from outside. The only outlet for the oil fumes was through the range hoods. At least 5–10 frying activities were conducted during indoor air monitoring at kitchen. Indoor air quality at control room was conducted in the office premises, which was a centrally air conditioned room and a non-smoking zone.

The heating apparatus was Liquid Petroleum Gas (LPG) stoves in the kitchen. The range-hood chimney used in the kitchen was a “low side wall” type. The kitchen floor was cleaned regularly during working hours with germicidal lotions. The work station of the kitchen was cleaned after each dish preparation. Kitchen workers wear aprons and head-coverings in the kitchen. They also washed their hands with antiseptic soap solution before and after each cooking activity. The water used in the kitchen was Reverse Osmosis (RO)-purified. Dishes were cleaned in a dish washer in a separate room away from the kitchen premises. Kitchen workers prepared vegetarian and non-vegetarian dishes using refined oils viz., sunflower oil and mustard oil.

### Indoor air quality monitoring for PM and TVOC

The indoor air concentration of particulate matter (PM_1_ and PM_2.5_) during working hours was determined using portable particulate matter measuring equipment (HAZ-DUST, Model EPAM-5000, Environmental Devices Corporation, New Hampshire, and USA) at kitchen and control room. The Total Volatile Organic Compounds (TVOCs) at the kitchen was monitored using a portable, handheld VOC monitor (MiniRAE 3000, RAE Systems, San Jose, California, USA). QA/QC procedures are maintained as per the instruction manual of the instruments. Pre and post checking of the flow rate before and after air monitoring was determined using flow meter. Pre and post–zero checking of the HAZDUST monitor was carried out. Pre and post–zero (indoor air without detectable contaminants) calibration checking of the VOC monitor was also carried out before and after air monitoring.

### Heat Stress

The temperature and humidity were monitored in the commercial kitchen and control area (office room of workers). Temperature was monitored using Indoor Air Quality Monitor (IQM 60, Aeroqual, Auckland, New Zealand). Pre and post–zero calibration checking of the indoor air quality monitor was also carried out before and after air monitoring. Relative humidity (RH) percentage was calculated from measurements of wet bulb and dry bulb thermometer readings using Hygrometer (LYNX, India). Humidex (humidity index) was calculated to describe how hot the weather feels to the average person, by combining the effect of heat and humidity [27] and compared with acceptable indoor air quality limits (Temperature and Relative humidity) [28].

### Indoor air sampling for PAHs

Indoor air sampling at the kitchen and control room was conducted with the sampler heads located at 0.5m from the pan and 1.5 m above the ground to simulate the human’s breathing zone. Air samples were collected using constant flow motor pump (General Electronic AC Motor, Fortwayne, Indiana, USA) operated at 10 L/min and equipped with a glass microfiber filter paper (Whatman, Cat No.1820 866) in the sampling head. The pump and sampling head were connected by a silicone tube. Pre and post checking of the flow rate was determined using flow meter. Sampling devices were protected against light during and after sampling by wrapping them in aluminum foil. Samples were taken to the laboratory in a black plastic bag, and kept in a -20°C refrigerator until analysis.

### Analysis of PAHs in indoor air filter paper sample using ultra high performance liquid chromatography (UHPLC)

The glass microfiber filter paper (Whatman, Cat No.1820 866) were weighed and extracted with 5 mL mixture of acetone and *n*-hexane (1:1, *v/v*) in dark condition using ultrasonic extractor for 30 min. The extracted solution was evaporated to dryness under gentle flow of nitrogen and reconstituted with HPLC grade acetonitrile up to 1.0 mL. Before injection, the extracted samples were filtered through PTFE membrane (pore size: 0.45μm, Millipore). Solvent blanks were also prepared in triplicate. The quantification of 16 PAHs were performed using UHPLC with photodiode array (PDA) detector (Nexara SR, Shimadzu Corporation, Kyoto, Japan) using a reported procedure [29] with a slight modification. Precisely, the chromatographic separation was performed using an analytical column (ZORBAX, Extend—C18 (2.1×150mm) 1.8μm size, Agilent Technologies, CA, United States). Acetonitrile-water mixture (80:20, *v/v*) was used as mobile phase with a flow rate of 0.4 mL/min at ambient temperature. Samples of 2μL were injected into the column. An isocratic elution conditions were used with a total run time of 13min per sample. The peak responses were obtained at a wavelength of 254 nm for all the samples. The data was processed with LC solution software (Shimadzu Scientific Instruments, Kyoto, Japan) for peak integration and quantification. Limit of detection and limit of quantification of all the 16 PAHs were found to be in range of 0.02–0.44 μg/mL, 0.08–1.44 μg/mL respectively.

### Urinary dipstick test analysis

Each subject submitted a fresh, random, mid-stream urine sample for the analysis, which was collected in a sterile container after completion of one day’s work activity at kitchen. Samples were taken to the laboratory in a black plastic bag, and kept in a -20°C refrigerator until analysis. Dipstick urinalysis was conducted using Clinitek Microalbumin 2 strips (Siemens Healthcare Diagnostics ltd, Frimley, UK) for microalbuminuria and URS 10 (Biosense Technologies, India) strips for specific gravity, with a U-Check Analytics sensor (Biosense Technologies Pvt. Ltd, Mumbai, India) for the analysis. The World Health Organization guidelines for urine sampling in occupational monitoring was followed in the study i.e., if a urine sample has creatinine concentration >300mg/dL or <30 mg/dL it should be considered invalid or to be re-sampled [30].

### Extraction of PAHs metabolites in urine samples of kitchen workers

Urine sample of kitchen workers were extracted with a procedure reported by Gupta *et al*., 2015 with slight modification to improve the detection limits of the method and reduce the matrix effect [31]. The urine samples (5mL) were deconjugated with 1 mL of 5N HCl for 30 min at 70°C. After that we add 15mL of Milli-Q water in deconjugated urine sample and pH of urine sample was adjusted to 6 with 5 N NaOH. An aliquot of 2 mL of extracted sample was taken after centrifugation in a 15 mL centrifuge tube. To this mixture of dispersive and extraction solvent [EtOH (300 μL) + TCE(100 μL)] was rapidly injected to form a cloudy solution. After centrifugation at 5000 rpm, allow for 5 minute to settle down the high-density extraction solvent to the bottom of the centrifuge tube (sediment phase).

The sediment phase of TCE formed was collected by using 100 μL syringe (Hamilton, USA) and the collected phase was transferred into a GC–MS auto sampler vial. The quantification of PAH metabolites *viz*., 1-napthol (1-NAP), 9-hydroxyfluorene (9-HF), 3-hydroxyfluorene (3-HF), 2-hydroxyfluorene (2-HF),9-phenanthrol(9-PHN), 1-hydroxypyrene (1-OHP) were performed using calibration graphs plotted between concentration and peak area of spiked PAH metabolites in controlled urine sample in the range of 0.2–200 ng/ mL.

### Analysis of urinary PAHs metabolites using Gas Chromatography-Mass Spectroscopy (GC-MS/MS)

GC–MS-MS analysis were performed using Thermo Scientific Trace GC Ultra gas chromatograph with a TriPlus autosampler (used for auto-IPS) coupled to TSQ Quantum XLS triple quadrupole mass spectrometer (Thermo Scientific, FL, USA). The TriPlus autosampler was operated in internal standard mode and BSTFA+TMCS (99:1, *v/v*) were kept at internal standard position in autosampler tray [30]. The injection of 2 μl of BSTFA +TMCS (99:1, *v/v*) and 2 μL of sample was performed in a single instance in programmed temperature vaporization splitless (PTV-SL) mode starting from 50°C (for 0.15 min) which was increased to 300°C at a rate of 1.8°C/s. Initial splitless time of 2 min was used followed by a split of 50 mL/min. Separation was carried out on TG-5MS capillary column (30 m length × 0.25 mm I.D. × 0.25 mm film thickness of 5% phenyl and 95% methyl polysiloxane)[30]. Carrier gas flow rate (helium) was maintained at 1 mL/min, injection were performed at 100°C (hold for 2 min), increased to 210°C at a rate of 15°C/min and finally reached to 280°C (hold for 5 min) at a rate of 10°C/min. Transfer line temperature and ion source temperature were kept at 290 and 220°C, respectively [30]. The mass spectrometer was operated in the electron ionization (±EI) mode at an electron energy of 70 eV. A solvent delay of 6 min was used. Full scan mode was used in the mass range of 50–450 amu [30]. After confirmation, the PAH metabolites were subjected to product ion scan, which facilitated the selection of selected reaction monitoring (SRM) transitions, which were further used for quantification of PAH metabolites [30].

### Quality assurance and quality control

For each batch of 15 samples analyzed, method blank, matrix-spiked samples were processed in triplicate. The intra- and inter-day precision of the method was assessed and percent relative standard deviation (%RSD) were found to be in the range of 2.6–4.9 and 6.1–8.9 respectivley and recoveries for PAH metabolites by GC-MS/MS were found to be in the range of 91–95%. The reported concentrations in samples were subtracted from blank values for all metabolites *viz*. 1-NAP, 2-HF, 3-HF, 9-HF, 9-PHN, 1-OHP. Method shows good linearity with regression coefficient (*R*^*2*^*)* values ranged from 0.991 to 0.998. Limit of detection and limit of quantification of all the PAH metabolites (1-NAP, 9-HF, 3-HF, 2-HF, 9-PHN, 1-OHP) were in the range of 0.06–0.6 ng/mL, 0.2–2 ng/mL respectively. The creatinine corrected urinary metabolite concentrations were used in the study as per World Health Organization guidelines [30] and mentioned in the earlier reference [32].

### Statistical analysis

Group means for age, height, weight, BMI, indoor air quality parameters and urinary specific gravity among kitchen workers and controls were compared using Student’s t-test. Frequencies for the prevalence of microalbuminuria among kitchen workers and controls were compared using the chi square test. All the urinary PAH metabolites detected above LOQ range (0.2–2 ng/mL) were included for statistical analysis. The concentrations of urinary PAHs are presented as their mean and median values, with 95% CI for mean. The criterion for statistical significance was set at *p* < 0.05. All the statistical analysis was performed using STATA software (IC 13, StataCorp LP, TX, USA).

## Results

**Fig 1:**
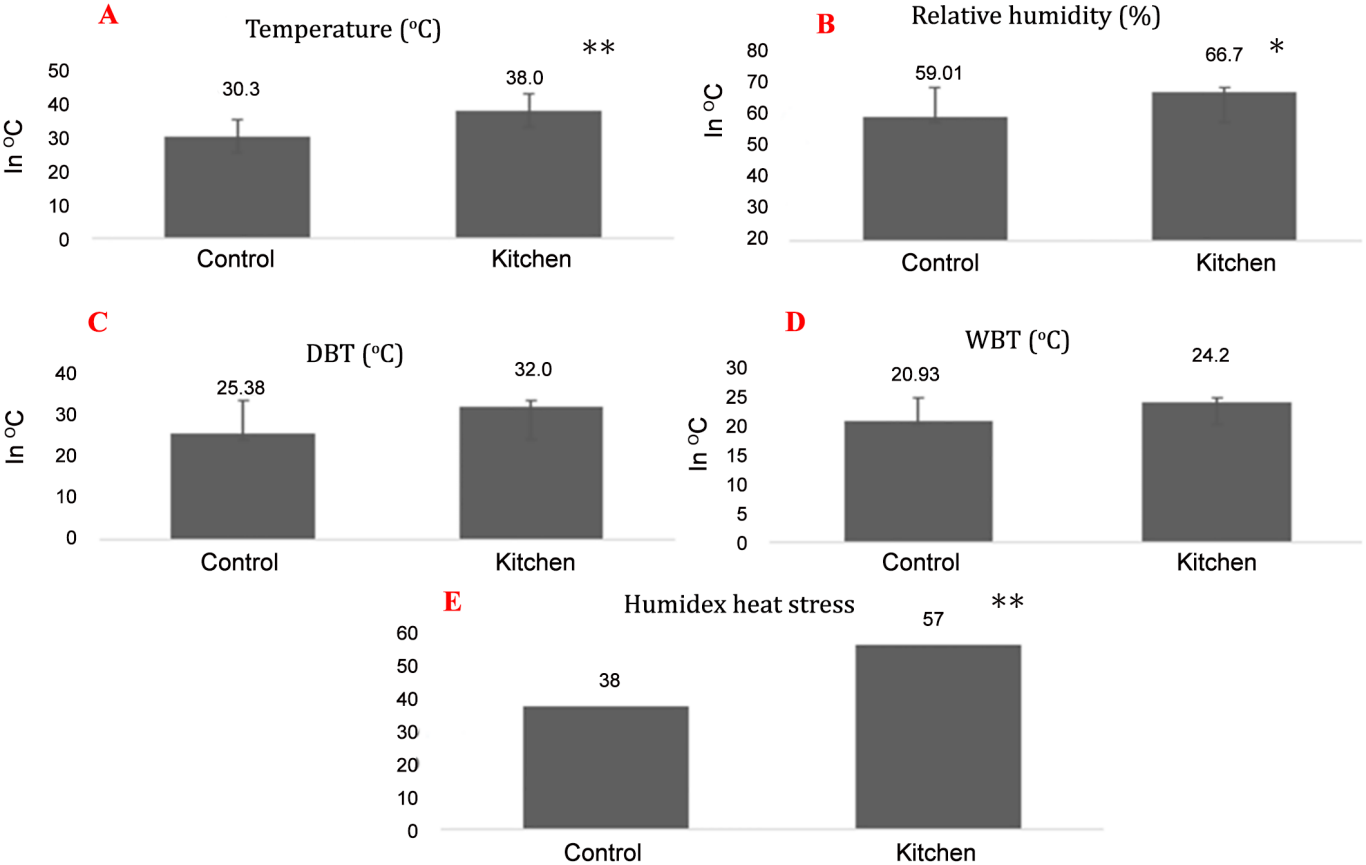
Humidex heat stress at kitchen and control room. (A) Temperature [Normal range: 20–27°C [28]; (B) Relative Humidity (%) [Normal range:30–60% [28]; (C) Dry bulb temperature (DBT); (D)Wet bulb temperature; (E) Humidex heat stress difference at control room and kitchen [Normal range:<29 [27]; *(p,0.05);**(p,0.001).

**Fig 2:**
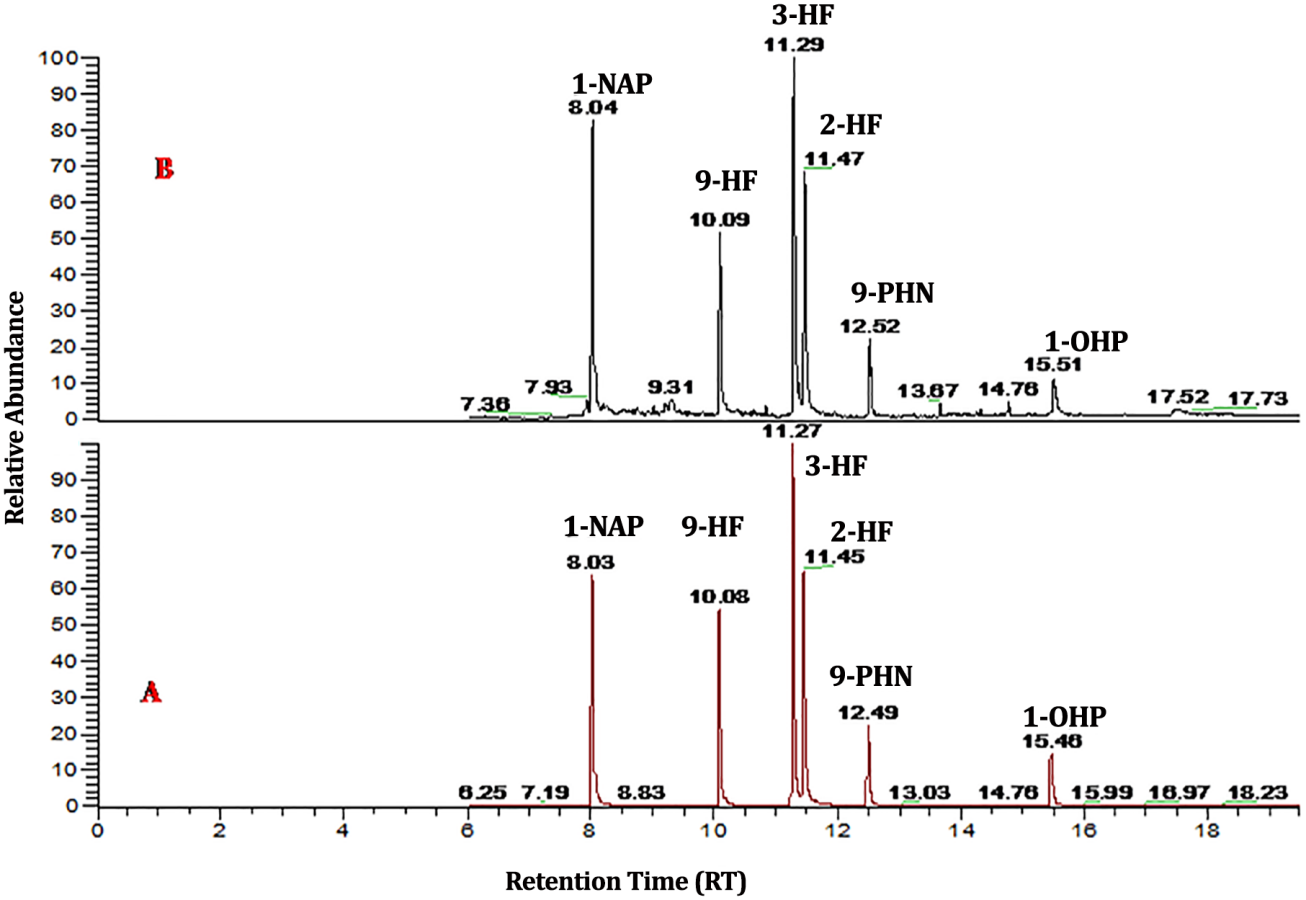
GC-MS/MS chromatogram of standard PAH metabolites mixture and exposed urine sample. (A)Standard mixture of PAH metabolites; (B)PAH metabolites found in sample (F-6) by GC-MS/MS (1-napthol (1-NAP), 9-hydroxyfluorene (9-HF), 3-hydroxyfluorene (3-HF), 2-hydroxyfluorene (2-HF), 9-phenanthrol(9-PHN), 1-hydroxypyrene (1-OHP).

Kitchen workers and their control counterparts had similar physical characteristics (Table 1). No kitchen worker was found to consume water during the working hours, other than during food intake time. High indoor air temperature and relative humidity was observed in the kitchen as compared to the control area (Fig 1). The heat generated in kitchen based on humidex plan was higher compared to control area (Fig 1). The specific gravity of urine was significantly higher (*p*<0.001) among kitchen workers compared to the control group (Table 2). The kitchen workers with higher urinary specific gravity range (1.02–1.03) were higher compared to lower specific gravity range (1.00–1.01) (Table 2). Significantly higher prevalence (80 cases– 85.11%) of microalbuminuria cases were observed among kitchen workers (*p*<0.001) compared to their control subjects (Table 2). PM_1_, PM_2.5_ and TVOC in indoor air were significantly higher in kitchen than the control room (Table 3). Indoor air PAHs identified was Napthalene, Fluorine, Acenaphthene, Phenanthrene, Pyrene, Chrysene and Indeno[1,2,3-cd]pyrene (Table 4). Concentrations of all PAHs in indoor air were above the Occupational Safety and Health Administration (OSHA) Permissible Exposure Level (PEL). A GC–MS/MS chromatogram of standard PAH metabolites mixture (500 ng/mL) and exposed urine samples in SRM mode are shown in Fig 2. Higher Urinary PAHs metabolites concentration for 1-NAP, 9-HF, 3-HF, 2-HF, 9-PHN and 1-OHP was observed in kitchen workers compared to controls (Table 5). No statistically significant difference in urinary PAH concentration was found between kitchen workers and controls. This may be due to large variation among the individuals in these groups.

**Table 1:** Physical characteristics of kitchen workers and controls.

Variable	Control (n = 94)	Kitchen workers (n = 94)	p value
**Age (yrs)**	31.7±9.4 (19–69)	32.0±8.3 (18–53)	p = 0.8
**Height (cm)**	166.5±6.5 (150–180)	164.8±6.8 (150–182)	p = 0.1
**Weight (kg)**	65.0±12.4 (43.6–109.5)	63.1±10.1 (44–86)	p = 0.2
**Body Mass Index (kg/m**^**2**^**)**	23.4±3.9 (16–34.6)	23.1±3.1 (16.5–30)	p = 0.7

Data are mean ± standard deviation (range).

**Table 2:** Urinary specific gravity and albumin/creatinine ratio (ACR) among kitchen workers and controls.

Parameters	Control (n = 94)	Kitchen workers (n = 94)	p value
**Urinary specific gravity**	1.01±0.01 (1–1.03)	1.02±0.01 (1–1.03)	p<0.001
**Microalbuminuria (MAU)**			
**ACR < 30**	73 (77.7)	14 (14.9)	
**ACR >30–300**	21 (22.3)	80 (85.1)	p<0.01

Data are mean ± standard deviation (range) or number (%). Abbreviations: ACR: Albumin creatinine ratio; MAU: Microalbuminuria.

**Table 3:** PM and TVOC concentration in indoor air at kitchen and control room.

Parameter	Control room	Kitchen	p value
**PM**_**1**_ **(μg/m**^**3**^**)**	26.6±14.2 (10–57)	71.9±48.4 (1–293)	p<0.001
**PM**_**2.5**_ **(μg/m**^**3**^**)**	34.7±12.6 (14–48)	81.3±59.2 (1–187)	p<0.001
**TVOC (μg/m**^**3**^**)**	0.08±0.04 (0.02–0.2)	1.26±0.35 (0.7–2.3)	p<0.001

Data are mean ± standard deviation (range). Abbreviations: PM_1_: Particulate Matter (1 micron); PM_2.5_: Particulate Matter (2.5 micron); TVOC: Total Volatile Organic Compound.

**Table 4:** Characterization of PAHs and their concentration (mg/m^3^) in indoor air at kitchen.

PAHs	Value
^#^OSHA norms (PEL OF PAHs)	0.2 mg/m^3^
Napthalene	3.1±0.1 (3.1–3.2)
Fluorene	0.81±0.1 (0.7–0.9)
Acenaphthene	17.71±2.33 (16–20.4)
Phenanthrene	0.21±0.01 (0.19–0.2)
Pyrene	6.1±1.1 (5.5–7.3)
Chrysene	0.2±0.03 (0.1–0.3)
Indeno [1,2,3-cd]pyrene	3.1±2.4 (0.3–4.8)

Data are mean ± standard deviation (range). Concentration of PAHs in kitchen air (Naphthalene; Fluorene; Acenaphthene; Phenanthrene; Pyrene; Chrysene; Indeno [1,2,3-cd]pyrene) were more than OSHA norms Permissible exposure limit in indoor air. Abbreviation: PAH: Polyaromatic hydrocarbon;

^#^OSHA: Occupational Safety and Health Administration; PEL: Permissible Exposure Level. Note: No PAHs were detected from control room

**Table 5:** Concentration of Urinary PAHs metabolite in creatinine adjusted urine samples (ng/ml) in study subjects.

PAHs metabolites	Control (n = 94)	Kitchen workers (n = 94)
1-NAP	4.10/2.15 (0.36–7.85)	10.69/5.86 (8.37–13.01)
9-HF	0.36/ND (0–0.97)	1.44/ND (1.0–1.89)
3-HF	0.83/0.98 (0.19–1.47)	2.60/1.12 (1.90–3.32)
2-HF	1.22/1.45 (0.3–2.13)	3.55/1.58 (2.60–4.51)
9-PHN	0.29/ND (0–0.79)	0.98/0.63 (0.68–1.27)
1-OHP	0.38/ND (0–1.46)	3.93/2.76 (3.11–4.75)

Data are mean/median. Values in brackets represent 95% CI for mean. Abbreviations: ND: Not detected; 1-NAP: 1-napthol; 9-HF: 9-hydroxyfluorene; 3-HF: 3-hydroxyfluorene; 2-HF: 2-hydroxyfluorene; 9-PHN: 9-phenanthrol; 1-OHP: 1-hydroxypyrene.

## Discussion

The present study is the first report on kidney dysfunctions among kitchen workers who have to endure thermally stressful conditions at their work place. The urinary specific gravity is considered as a reliable biomarker for heat stress among workers and was used to study the health effects of kitchen environment where heat levels were significantly high. PAHs and VOCs can bind with fine PM particles in indoor air and enter the body through inhalation, ingestion and dermal contact. This can result in inflammatory responses in kidney which can be the aetiological factor for higher prevalence of microalbuminuria observed among kitchen workers. In this study all the PAHs detected in the indoor air were metabolized to their respective metabolites and detected in urine samples. The association between multiple physical and chemical pollutants like heat, PAHs, VOCs and PM exposure in indoor air of kitchen and kidney dysfunctions among kitchen workers was evidenced from this study. Particle emissions from frying include those from the stove (electric or gas), pan, frying oil, food that is being cooked, water vapor emitted from the food and possible reactions between food and oils at the temperature of frying / cooking [33, 34].

As per the earlier report [31] “In commercial kitchens, PAHs came from two sources, cooking practice and oil-fumes, however the cooking practice had a more predominant contribution to PAHs in commercial kitchen air. In domestic kitchens, except for cooking practice and oil-fumes, there were other PAHs sources, such as smoking and other human activities in the domestic houses, where 3–4 ring PAHs mainly came from cooking practice (4 ring PAHs (PYR,CHRY) and six rings (Indeno[1,2,3-cd] pyrene)”. NA, ACE (2-ring PAHs) was the most predominant kind, mostly resulting from the evaporation of mothball containing a large quantity of NA, used to prevent clothes against moth. A fingerprint of oil-fumes was the abundance of 3-ring PAHs (PHN,FLU) [35].

Our study findings support an earlier report which showed an increase in specific gravity of urine of sugarcane cutters due to heat stress at their work place [36]. The kitchen workers in the present study are exposed to extreme heat during cooking activities. The heat exposure together with the wearing of tight aprons leads to sweating and dehydration among workers. The regular exposure to these conditions may have resulted in higher urinary specific gravity. Restaurant employees who are regularly subjected to extreme heat are prone to heat rashes, heat cramps, heat exhaustion and heat stroke [37].

Albumin excretion in urine is considered as an early indicator of kidney dysfunction among workers occupationally exposed to extreme heat at work place [38]. Microalbuminuria and increased urine albumin to creatinine ratio are regarded as early predictors of kidney damage [39]. Renal function marker (creatinine) rises in subjects who are affected by heat stress [40]. Therefore, it is proposed that higher indoor air temperature and relative humidity during cooking activities and poor ventilation in canteen has led to heat stress-related kidney dysfunctions among kitchen workers in the present study. Apart from a rise in indoor air temperature in the kitchen, there is a simultaneous increase in humidity, which can lead to hyperthermia among workers, as proposed in an earlier report [41]. Lack of efficient exhaust facility and ventilation hood at canteen may be one reason for the inability to flush out the indoor hot and humid air.

Heat stress causes urinary volume depletion and can cause acute kidney injury [41]. The higher concentration of urine among worker indicates insufficient hydration [36]. Hydration status was estimated from urinary specific gravity, which is considered to be an important indicator of the absolute hydration status of the body and relative changes in hydration status over time [42]. Hyperthermia-induced urine volume depletion leads to repeated sub-clinical damage to the kidney, and further progresses to CKD [43, 44]. Hot environmental condition during cooking activities causes hyperthermia. Most of the kitchen workers in tropical countries are exposed to the thermal stress of kitchen due to consumption of insufficient water at work place. They consequently suffer from urine volume depletion, further causing changes in blood perfusion and leading to ischemic injury of the kidney [41, 45].

The combination of food added to the oil affects the particle size distribution [34, 46]. A linear trend between level of exposure to particulate matter and measures of poor kidney function viz., albumin-to creatinine ratio, was demonstrated in an earlier study [47]. Participants with the highest exposure to particulate matter had significantly worse kidney function than those with low exposure. This report [47] supports our observation of higher PM concentration in indoor air and its etiological association with poor albumin/creatinine ratio and further higher prevalence of microalbuminuria cases.

There is opportunity for exposure to a number of chemicals in the workplace or ambient environment that are possible nephrotoxicants [48]. The role of multiple toxicants viz., PM, PAHs, VOCs and extreme heat (indoor air quality monitoring in the kitchen) as etiological factors that may contribute to renal failure is so far not clear. Our study suggests these multiple toxicants like PM, VOCs and PAHs in indoor air and PAH exposure (as measured by urinary levels of PAH metabolites) can be considered as etiological factors for microalbuminuria in kitchen workers. Earlier studies on coke oven workers, aluminum smelter workers, foundry workers, road pavers and the psoriasis patients topically treated with coal tar showed higher levels of urinary hydroxylated PAH metabolites in the occupationally exposed workers than in the controls [21–25, 49]. Similar observation was observed among the kitchen workers in the present study. 1-Hydroxypyrene is the urinary marker most commonly used for assessing human exposure to PAHs. Positive correlation was found between the increase of urinary 1-hydroxypyrene and the airborne PAH exposure in the occupationally exposed workers [23, 24].

It is not clear whether the lower background exposure to PAHs can be associated with inflammatory effects in humans. In an earlier study, a positive association between urinary 1- hydroxypyrene with total WBC count (serum markers of inflammation) in males compared to females was reported [50]. An association was reported earlier between chronic inflammations with microalbuminuria [51]. In this context, our study proposes that PAH exposure related inflammatory response may have resulted in the microalbuminuria among kitchen workers. Acute kidney injury and subclinical damage like microalbuminuria, as observed in the present study can lead to chronic kidney disease (CKD) in the absence of functional recovery from the initial injury [41].

In India, a wide variety of occupations including work in the kitchens of food industry is performed under adverse and unhealthy conditions [52]. Environmental and occupational exposure may cause renal tubular and glomerular impairments [38]. Large population studies have consistently shown that albuminuria and proteinuria can strongly and independently predict the risk of chronic kidney disease progression [53, 54]. Occupational heat stress, VOCs and PAHs contamination in indoor air at kitchen requires more attention, especially among kitchen workers, considering the high temperature and humidity at work place and its consequences like microalbuminuria.

As reported earlier [55] the *ad libitum* availability of water “on the job” in personal water bottles should be mandatory at work place. The report [55] also stresses the need for workers to be educated about the need to drink small amounts of water frequently (the recommended value during their induction and annual refresher training is 250 mL every 15 minutes). The present study proposes regular heat stress monitoring in the kitchens of food industry/canteens and adopt appropriate engineering control measures to reduce heat inside kitchens, as a mandatory requirement. Training and education on heat stress management should be provided to the kitchen workers by the authorities. Also the present study findings need replication in other locations with adequate sample sizes.

In earlier studies, the samplers were placed near the exit of the exhaust duct at the roof of the restaurants/hotels/canteens, which convey the source apportionment of kitchen air pollutants to the ambient air thus missing indoor air samples from the breathing zone of kitchen workers. Moreover, most of the earlier reports on indoor air quality observations fail to correlate with health effects of the kitchen workers. One of the strength of present study was the indoor air quality monitoring in kitchen at breathing zone of kitchen workers. Thus, the workers are directly exposed to heat as well as PM, VOCs and PAHs generated from kitchen. There is wide variation between the minimum and maximum concentration measured for fine PM particles, which can be related to the frequency of food preparation including frying and other cooking activities. Medication, diet and habits create additional variability that would greatly hinder the identification and concentration of urinary metabolite profile in humans [56].

The study has limitations as well. The cross sectional nature of the study does not allow us to draw temporal or causal inferences regarding multiple indoor air pollutants and kidney dysfunctions. Urinary PAH biomarker measurements reflect recent exposure to PAHs and do not reflect differences between current exposure sources and past exposure sources for each subject. These biases are likely to be non-differential biases, which would minimize any associations observed. There is a need to replicate these findings in future prospective studies with adequate sample size.

Commercial kitchen managers and owners can follow the tips mentioned below to reduce their employees’ susceptibility to heat-related dangers proposed by Occupational Safety and Health Administration [57].

- Give employees time to adapt at the work place, prior to taking up the job.
- Awareness about the danger of heat stress should be imparted to kitchen workers.
- Encourage workers to drink plenty of water.
- Keep cooking areas as cool as possible.
- Make sure the vent hoods are working properly.

In conclusion, continuous heat exposure in kitchens due to cooking can alter kidney functions *viz*., high specific gravity of urine in kitchen workers. Exposure to PM, VOCs and PAHs in indoor air and body burden of these toxicants may lead to inflammation which can cause microalbuminuria in kitchen workers, as observed in the present study. Better ventilation strategies should be implemented as an intervention step to reduce the heat and air pollutants in kitchens. Also this study stresses the need to educate workers to consume sufficient water during work activities in the kitchen. This study augments an increasing body of evidence of kidney dysfunction among kitchen workers exposed to indoor air pollutants in kitchen.
